# Does left ventricular hypertrophy affect cognition and brain structural integrity in type 2 diabetes? Study design and rationale of the Diabetes and Dementia (D2) study

**DOI:** 10.1186/s12902-017-0173-7

**Published:** 2017-04-07

**Authors:** Sheila K. Patel, Carolina Restrepo, Emilio Werden, Leonid Churilov, Elif I. Ekinci, Piyush M. Srivastava, Jay Ramchand, Bryan Wai, Brian Chambers, Christopher J. O’Callaghan, David Darby, Vladimir Hachinski, Toby Cumming, Geoff Donnan, Louise M. Burrell, Amy Brodtmann

**Affiliations:** 1 The Florey Institute of Neuroscience and Mental Health, Melbourne Brain Centre, Austin Health, 245 Burgundy Street, Heidelberg, VIC 3084 Australia; 2 Department of Medicine, University of Melbourne, Austin Health, Level 7, Lance Townsend Building, 145 Studley Road, Heidelberg, VIC 3084 Australia; 3 Austin Health Endocrine Centre, Heidelberg, VIC Australia; 4 Department of Cardiology, Austin Health, Heidelberg, VIC Australia; 5 Department of Neurology, Austin Health, Heidelberg, VIC Australia; 6 Department of Clinical Pharmacology, Austin Health, Heidelberg, VIC Australia; 7 Department of Clinical Neurological Sciences, London Health Sciences Centre, University of Western Ontario, London, Canada

**Keywords:** Type 2 diabetes mellitus, Dementia, Alzheimer’s disease, Left ventricular hypertrophy, Dementia, Cognition, D2 study

## Abstract

**Background:**

Cognitive impairment is common in type 2 diabetes mellitus, and there is a strong association between type 2 diabetes and Alzheimer’s disease. However, we do not know which type 2 diabetes patients will dement or which biomarkers predict cognitive decline. Left ventricular hypertrophy (LVH) is potentially such a marker. LVH is highly prevalent in type 2 diabetes and is a strong, independent predictor of cardiovascular events. To date, no studies have investigated the association between LVH and cognitive decline in type 2 diabetes. The Diabetes and Dementia (D2) study is designed to establish whether patients with type 2 diabetes and LVH have increased rates of brain atrophy and cognitive decline.

**Methods:**

The D2 study is a single centre, observational, longitudinal case control study that will follow 168 adult patients aged >50 years with type 2 diabetes: 50% with LVH (case) and 50% without LVH (control). It will assess change in cardiovascular risk, brain imaging and neuropsychological testing between two time-points, baseline (0 months) and 24 months. The primary outcome is brain volume change at 24 months. The co-primary outcome is the presence of cognitive decline at 24 months. The secondary outcome is change in left ventricular mass associated with brain atrophy and cognitive decline at 24 months.

**Discussion:**

The D2 study will test the hypothesis that patients with type 2 diabetes and LVH will exhibit greater brain atrophy than those without LVH. An understanding of whether LVH contributes to cognitive decline, and in which patients, will allow us to identify patients at particular risk.

**Trial registration:**

Australian New Zealand Clinical Trials Registry (ACTRN12616000546459), date registered, 28/04/2016

**Electronic supplementary material:**

The online version of this article (doi:10.1186/s12902-017-0173-7) contains supplementary material, which is available to authorized users.

## Background

Type 2 diabetes mellitus is associated with an increased risk of dementia [1, 2] and more specifically, a two-fold increase in the risk of Alzheimer’s disease [3]. Many factors may contribute to this strong association, including increased stroke incidence, white matter microstructural changes, hyperinsulinaemia and metabolic syndrome, as well as the cross-association of mid-life obesity and type 2 diabetes [4]. A diagnosis of diabetes in midlife increases the risk of both vascular cognitive impairment and Alzheimer’s disease [5].

Magnetic resonance imaging (MRI) markers of structural brain aging such as reduced total brain volume, cortical thickness, hippocampal volume, and increasing white matter hyperintensity load, are correlated with performance on neuropsychological tests and are powerful predictors of dementia [6, 7]. A strong association between midlife vascular risk factors and structural brain aging has been demonstrated [8], although longitudinal brain volume changes have not been thoroughly examined in patients with type 2 diabetes. The PROSPER study findings suggested that elderly non-demented patients with type 2 diabetes had accelerated progression of brain atrophy compared to non-diabetic individuals and provided evidence for the hypothesis that diabetes exerts deleterious effects on neuronal integrity [9].

There is little evidence that intensive glycaemic management as reflected by lower HbA_1c_ levels in type 2 diabetes is associated with reduced dementia risk [10, 11] or cognitive decline [12]. The ACCORD-MIND study investigators [10] found no evidence that an intensive glycaemic treatment strategy targeting HbA_1c_ to <6% altered cognitive outcomes in patients with type 2 diabetes. However, the ACCORD study was halted due to higher mortality rates from cardiovascular events in the intensive arm [13]. Subsequent follow-up of the ACCORD-MIND participants found an association between poor cognitive function and the risk of severe hypoglycaemic events [14]. A further 80 months of follow-up in the ACCORDION MIND trial showed no long term benefits of intensive glycaemic control on cognition or brain MRI outcomes [15]. It therefore remains unclear if tight glycaemic control as reflected by lower HbA_1c_ levels lead to lower risk of dementia and better biomarkers are needed for the prediction of cognitive decline to stratify the risk of dementia in type 2 diabetes.

Patients with type 2 diabetes have associated hypertension [16], a high prevalence of previously unknown heart failure [17] and left ventricular dysfunction [17, 18]. Left ventricular hypertrophy (LVH) is also highly prevalent in patients with type 2 diabetes [18] and hypertension [19] and is an established independent predictor of adverse cardiovascular outcomes, including stroke [20] and heart failure [21]. Patients with type 2 diabetes and co-morbid hypertension have increased left ventricular mass, more concentric left ventricular geometry, lower myocardial function, that is independent of age, sex, body size, arterial blood pressure and atherosclerosis, compared to hypertensive individuals without type 2 diabetes [16]. Diabetes and hypertension predispose to the ischemic small vessel disease underlying lacunar infarction. Lacunar infarction can result in cognitive decline from selective damage to frontal-subcortical circuits subserving processing speed and executive function, and these deficits are often observed in type 2 diabetes [22].

There is a strong association between cardiac disease and impairment across many cognitive domains, particularly in tests of executive function [23]. Heart failure is strongly associated with cognitive impairment [24–26] and is an independent risk factor for the development of Alzheimer’s disease and cerebrovascular disease [16]. Carotid atherosclerosis is associated with cognitive impairment [27], measures of structural brain aging [8] and reductions in parietal gray matter [8]. LVH is associated with progressive cognitive decline, independent of blood pressure and large artery stiffness [28]. Left ventricular mass is associated with worse performance on tests of both visuospatial and verbal memory [29], and there is a nonlinear association between left ventricular ejection fraction (EF) and measures of accelerated structural and cognitive brain aging [30].

### The D2 (diabetes and dementia) study

The diabetes and dementia study (D2) study is an observational study in which we will intensively study the cardiovascular status of patients with type 2 diabetes and investigate if LVH predicts cognitive function, or if specific cardiac indices predict cognitive decline and brain atrophy. We will recruit participants with type 2 diabetes and integrate transthoracic echocardiography, carotid imaging, brain imaging with MRI, circulating and genetic biomarkers, and neuropsychology tools, to establish the relationship between type 2 diabetes, brain atrophy, and cognitive decline in a cohort of 168 individuals with and without LVH. At baseline, all participants will have structural neuroimaging with MRI to assess global brain volume, regional cortical thickness and hippocampal volume, a 12 lead electrocardiogram (ECG), an echocardiogram to assess the absence or presence of LVH, a carotid ultrasound to assess vascular disease, 24-h ambulatory blood pressure and ECG, cognitive assessment, and apolipoprotein E (*APOE*) gene risk assessment. All of the aforementioned assessment tools will be repeated at the 24-month follow up period.

## Methods

The D2 study has been approved by the Human Research Ethics Committee at Austin Health (HREC/15/Austin/490) and is registered at the Australian New Zealand Clinical Trials Registry (ACTRN12616000546459). Funding is provided through the National Health and Medical Research Council of Australia (APP1094974). Informed written consent is obtained from the participants prior to any data collection.

### D2 Study design

The D2 study is a single center, observational, longitudinal, case-control study. One hundred and sixty-eight adult patients, aged greater than 50 years with type 2 diabetes will be followed: 50% with LVH (case) and 50% without (control). Patients will be assessed at baseline (0 months) and will return for tests at the 24 month time-point. Based on recruitment in our other diabetes, cognitive and imaging trials, it is expected that 80–90 patients will be recruited annually for the first 2 years. Recruitment of new patients will be completed at the end of 2 years, allowing the next 2 years for follow-up.

### Study hypotheses

1. Patients with type 2 diabetes and LVH will exhibit greater brain atrophy as assessed by MRI structural changes than those without LVH.
2. Brain atrophy as assessed by MRI structural changes will be associated with worse cognitive performance.
3. Increased left ventricular mass with specific left ventricular geometric patterns will be associated with the most severe regional brain atrophy and cognitive decline.

### Study aims

1. To establish whether diabetic patients with LVH have increased rates of brain atrophy and cognitive decline.
2. To establish whether the specific LV geometric measures are associated with these brain volume changes.

### Study outcome measures

Our primary endpoint is brain volume change at 2 years (hypothesis 1). Our co-primary outcome is the presence of cognitive decline at 2 years (hypothesis 2). We shall use advanced brain imaging to accurately measure brain volume and correlate this with cardiac echocardiography, carotid intima media thickness (CIMT), and 24 h blood pressure and ECG monitoring in 168 patients with type 2 diabetes, with longitudinal testing allowing measurement of change at 24 months. Concurrent neuropsychological testing will indicate whether the clinical profile of cognitive impairment and dementia aligns with measures of brain volume.

### Eligibility criteria

Inclusion criteria are as follows: (1) aged over 50 years; (2) no prior neurological or psychiatric disease, including stroke; (3) ability to give informed consent and participate in cognitive testing. The study exclusion criteria are as follows: (1) known prior stroke, transient ischemic attack or dementia; (2) significant medical co-morbidities precluding participation in cognitive testing, or making survival for 24 months unlikely; (3) normal exclusion criteria for MRI; e.g., implanted metal, severe claustrophobia; (4) severe diabetic nephropathy: estimated glomerular filtration rate (eGFR <30 mL/min/1.73 m^2^).

### Study assessments

The schedule of assessments is provided in Table 1. Testing will include the following: a pre-study screening questionnaire and MRI safety check to determine eligibility; demographic and medical history questionnaire; clinic measurement of height, weight, waist and hip; echocardiogram, ECG; CIMT; 24 h ambulatory blood pressure and ECG monitor; cognitive assessment; MRI scan; physical activity monitor; and a blood sample (to determine *APOE* genetic risk and analyses of genetic and plasma/serum biomarkers).

**Table 1:** Schedule of assessments at baseline (0 months) and 24 months

Assessments	Brief description	Time-point (months)	
		0	24
Pre-study screening questionnaire (to determine study eligibility)	Study eligibility criteria	✓	
MRI safety check questionnaire	MRI eligibility	✓	✓
Demographic and medical history questionnaire	Questionnaire completed with the study investigator	✓	✓
Weight, height, waist and hip measurement	Clinical assessment	✓	✓
Transthoracic echocardiogram	Assess cardiac structure and function	✓	✓
Blood sample	Plasma electrolytes, glucose, HbA_1C_, lipids and urinary analyses. Blood collected for *APOE* genotyping and assessment of other genetic and circulating biomarkers.	✓	✓
Carotid intimal medial thickness	B-mode ultrasound to assess left and right internal carotid arteries and the degree of stenosis	✓	✓
24 h ambulatory blood pressure and ECG Holter	Simultaneous measurement of blood pressure and ECG	✓	✓
MRI scanning on a Siemens 3 T Skyra scanner using a 64 Channel receive only head coil.	• High resolution isotropic T1 3D MPRAGE (structural scan)	✓	✓
	• High resolution isotropic T2 FLAIR 3D		
	• High resolution isotropic T2 3D		
	• Susceptibility-weighted image (SWI) for iron deposition		
	• Diffusion-weighted imaging (DWI)		
	• GRE field mapping		
	• BOLD 7 min free run (resting state connectivity)		
Cognitive and mood evaluation	Validated tools for assessing mood, global cognitive ability, attention, visuospatial ability, executive function, language, memory, processing speed	✓	✓
Physical activity assessment	SenseWear Armband (BodyMedia FIT)	✓	✓

### Details of questionnaire and measurements

#### Pre-study screening and MRI safety check questionnaires

A short questionnaire will be used to ask questions related to the study eligibility, which will be key to identifying patients that may be unsuitable for MRI testing. Those identified as unsuitable will be informed at this stage and no further measurements will be conducted. Eligible participants will complete a detailed MRI safety questionnaire with the investigator to obtain more specific information about their history of surgical procedures to determine whether they have implants that might not be suitable for a 3 T MRI scan. Participants will be excluded if they cannot have a research 3 T MRI scan (e.g., because they have a metal implant that has not been cleared for safety in a 3 T MRI scanner).

#### Demographic and medical history questionnaire

A questionnaire and medical record review will be conducted at the time of the echocardiogram and blood collection. The following information will be collected: age, sex, duration of diabetes, smoking history, alcohol consumption, current drug therapy and dose, socioeconomic status family history of cardiovascular disease, medical history including general health and co-morbidities (cardiac disease, hypertension, dyslipidaemia, the presence of micro-vascular disease (nephropathy, retinopathy, neuropathy) and other macro-vascular complications (peripheral vascular disease). The questionnaire will be cross-checked by medical record review at Austin Health, and from their clinical and specialist reports if recruited outside of Austin Health.

#### Physical measurements

Participants’ height and weight (to determine body mass index (BMI) and body surface area) will be assessed. Weight will be measured to the nearest 0.1 kg on standing digital scales without shoes and light clothing. Height will be measured using a wall-mounted stadiometer to the nearest 0.1 cm. Waist and hip circumferences will be measured using a spring loaded tape. Waist circumference is measured at the mid-point between the lower costal margin and the iliac crest. Hip circumference is measured over the greater trochanters.

#### Transthoracic echocardiography (TTE)

TTE will be performed by a cardiologist using a commercially available portable ultrasound system (Vivid i, GE Healthcare). Standard parasternal and apical views will be used to assess left ventricular mass. Measurements are obtained according to the American Society of Echocardiography (ASE) recommendations [31]. M-mode echo will be used to measure cardiac dimensions and wall thickness. Ejection fraction will be calculated using the modified Simpson’s rule. Left ventricular mass will be indexed using the ASE corrected convention, by dividing left ventricular mass by height^2.7^. LVH will be defined as >49 g/m^2.7^ for males and >45 g/m^2.7^ for females. Relative wall thickness will be calculated, and left ventricular remodelling classified as normal, eccentric, or concentric remodelling and concentric LVH.

#### Blood collection and measurements

Fasting plasma electrolytes, glucose, HbA_1C_, lipids and urinary analyses will be obtained on all patients at baseline and at 24 months. The D2 study will collect additional plasma, serum and whole blood for genetic analyses. The exploration of novel circulating biomarkers and genetic determinations that lead to LVH and/or brain atrophy will be conducted within our laboratory. Whole blood will be collected and divided between lithium heparin and EDTA tubes for plasma and genetic samples and a plain tube for serum. Serum will be extracted and aliquoted for storage at -80 °C until assayed. For plasma, the sample tube will be stored on ice and centrifuged within 15 min to separate the plasma. The plasma will be aliquoted for storage at -80 °C until assayed. Whole blood will be collected in a potassium EDTA tube and immediately stored at -80 °C until DNA extraction. *APOE* genotyping will be performed in our laboratory. Genomic DNA will be extracted using the Qiagen DNA Maxi kit (Qiagen, Germany) and the *APOE* region of interest will be amplified with specific primers. The resulting DNA fragment will be sequenced in the forward and reverse direction on an ABI 3130-xl genetic analyser (Applied Biosystems, CA, USA). Participants will be informed of their *APOE* ε4 status by the Principle Investigator and those with *APOE* ε4 homozygosity will be offered counselling via the Neurogenetics Clinic at Austin Health.

#### Carotid Doppler ultrasound

CIMT measurements will be conducted in the Neurology diagnostics laboratory, Austin Health by a single technician. High-resolution B-mode ultrasound images will be obtained on both right and left internal carotid arteries. CIMT will be measured, and the degree of stenosis will also be classified into accepted clinical grades for incorporation into multivariate analyses: none, <50%, 50–69%, 70–79%, 80–89%, 90–99% and occluded (NASCET criteria) [32].

#### Ambulatory blood pressure and ECG monitoring

Each participant will have a combined Holter monitor simultaneously assessing ambulatory blood pressure and ECG data (CardXplore, Meditech, Hungary).

#### MRI acquisition and processing

Scans will be acquired for gray and white matter anatomical, structural and functional connectivity and iron estimation analyses. High resolution structural MRI of study subjects will be acquired using a 3 T Siemens Skyra MR scanner. The following sequences will be acquired: T1-weighted three-dimension (3D) magnetization-prepared rapid gradient-echo (MP-RAGE) for anatomical analyses; susceptibility-weighted image (SWI) for iron estimation and microhemorrhage imaging; fluid-attenuated inversion recovery (FLAIR) to assess chronic white matter ischemic changes and diffusion weighted imaging (DWI) to assess white matter structural integrity.

Regional cortical gray matter atrophy will be assessed using vertex-wise cortical thickness analysis. Cortical thickness will be mapped using the FreeSurfer software package for analysis of structural MRI data (http://surfer.nmr.mgh.harvard.edu/fswiki) [33]. The automated cortical and subcortical parcellation routines also provided with FreeSurfer will be used to investigate regional volume changes. We will use processing streams developed specifically by our group for longitudinal studies [34, 35]. Hippocampal volume changes will be assessed using manual hippocampal segmentation. Iron deposition and cerebral microbleeds will be assessed with susceptibility-weighted image analyses. The MRI protocol is provided in the Additional file 1.

#### Cognitive and mood assessment questionnaire and tests

Participants will complete a neuropsychological assessment. The assessment will include several paper-and-pencil and computerized cognitive tasks and mood questionnaires. Six cognitive domains will be examined, including (i) attention, (ii) visuospatial ability, (iii) executive function, (iv) language, (v) memory, and (vi) processing speed. The tasks used to measure each cognitive domain are listed in Table 2. This protocol has been modelled on that validated by the CANVAS study, a longitudinal study of patients after stroke [36]. All tasks are designed for serial testing and are less affected by practice effects. Performance on individual cognitive tests will be standardised using established norms and domain scores will be calculated by averaging the standardised scores from each contributing test (e.g., Trail Making Test as a measure of attention). Assessment of mood will be completed using the seven-item Generalized Anxiety Disorder (GAD-7) questionnaire and the nine-item Patient Health Questionnaire (PHQ-9). These questionnaires are based on criteria for the diagnosis of generalised anxiety and depression (Diagnostic and Statistical Manual-Fourth Edition: DSM-V) [37].

**Table 2:** Tasks used to measure each cognitive domain

Cognitive domain	Cognitive task
Global Cognitive Ability	Montreal Cognitive Assessment [45]
	The Clinical Dementia Rating [46]
	National Adult Reading Task [47]
Attention	Trail-Making Test [48]
	Digit Span Task (WAIS-IV) [49]
	Identification Task (Computerised CogState battery) [50]
	One Back Task (Computerised) [50] CogState battery)
Visuospatial Ability	Rey Complex Figure Test (Copy) [51]
Executive Function	Clock Drawing Task – CLOX [52]
	Controlled Oral Word Association Test [53]
Language	30-item Boston Naming Test [54]
	Verbal Fluency Test [53]
Memory	Rey Auditory Verbal learning Task [55]
	Rey Complex Figure Test (Recall) [51]
Processing Speed	Detection Task (Computerized CogState battery) [50]
	Digit-Symbol Task (WAIS-IV) [49]

#### Physical activity monitoring

The SenseWear Pro2 Armband (HealthWear BodyMedia, Pittsburgh, PA, USA) is a portable device which enables monitoring of energy expenditure and activity. The device uses a two-axis accelerometer, a heat flux sensor, a galvanic skin response sensor, a skin temperature sensor, and a near-body ambient temperature sensor to capture data and will be worn by each participant on the upper arm for 7 days. The data will be converted into Metabolic Equivalents minutes (METs*minutes) and energy expenditure using an algorithm (Bodymedia, Sense Wear 6.1) [38]. The SenseWear monitor has been validated for measuring energy expenditure in diabetes against double-labeled water [39].

### Sample size

We aim to recruit 168 patients with type 2 diabetes. Patients will be divided based on the presence of LVH. Higher left ventricular mass and concentric geometric changes have been demonstrated in approximately 35–40% of patients with type 2 diabetes [16, 17]. Our data suggested more than 50% of participants will have LVH [18]. We calculated our sample size in order to include sufficient participant numbers for both the primary endpoint comparison (brain volume) and for the primary outcome measure (presence of cognitive impairment). The primary endpoint requires a direct comparison of two groups: type 2 diabetes patients with LVH and those without. We based our effect size on published brain volume and cortical thickness estimates performed on healthy controls, cerebrovascular disease patients and Alzheimer’s disease patients [40–42], and prior imaging of healthy controls and stroke patients performed by our group [43]. We use an average brain volume change of <0.5% per annum for controls, 1–2% for cerebrovascular disease, and 2–4% for dementia [44].

We used a modified retrospective case-control method. All participants have the exposure (type 2 diabetes), which will naturally halve by the additional risk factor (LVH, expected prevalence around 50%), but only some of them will develop the primary outcome (cognitive decline). We used an ANCOVA method to estimate sample size for four samples (cognitively impaired LVH, non-cognitively impaired LVH, cognitively impaired non-LVH, non-cognitively impaired non-LVH) with repeated measures, including a correlation score between baseline and follow-up. Using alpha = 0.05 (two-sided), power = 0.8 and an estimated correlation of 0.1, we estimated that 140 (i.e., 35 in each of 4 groups) participants will be required. Given we expect around 20% attrition due to death or non-participation (unable to participate in testing due to new pacemaker/implanted metal/other medical issue, lost to contact, no longer interested), we have estimated a total recruitment number of 168. This model has used the conservative estimates from these results and assumes the following: that 30% of patients will be cognitively impaired at 2 years; a 1% total annual brain volume change for patients with type 2 diabetes; that 30% of patients will exhibit specific left ventricular geometric changes; and a low correlation between baseline and follow-up measures.

### Statistical analysis

The analysis of this study will be performed in three parts as follows.

- ***Hypothesis 1: comparing brain volumes between participants with and without LVH***. Independent samples t-tests will be used to determine differences between participants with and without LVH at each time-point. Repeated-measures t-tests will be used to determine the extent of brain volume loss across the two time-points. These results will be compared between groups. In the cortical thickness analyses, the large number of statistical inferences being carried out across the cortical sheet (approximately 160,000 vertices per hemisphere) requires adjustment of the statistical threshold. This is to control for the increased number of false positive findings using false discovery rate correction procedure widely used for cortical thickness analyses. In the volumetric analyses, total brain volume, regional white matter, cortical and subcortical volume changes will be modelled using a repeated measures design. Intracranial volume, which is determined partially by gender and race, is a known confounding factor that contributes to regional brain volumes. This will be controlled for in the statistical analysis of regional brain volume change. We have demonstrated this to be a reliable index of brain volume in previous studies [43].
- ***Hypothesis 2: correlating brain volume with cognitive performance.*** The initial regressive analysis will examine differences between cognitively impaired and normal participants at 2 years. We will correlate both global and regional measures of brain volume with the groups. Global brain and regional hippocampal volumes, and mean regional cortical thickness values for the stated regions of interest will be compared between two groups, in order to correlate LVH-associated brain volume changes with cognition. We will also perform a multivariate analysis across the cohort as a whole to investigate whether there is a dose-related association with any extent of cardiac impairment. Multivariate regression will be conducted to determine whether brain volume loss correlates with the presence or absence of cognitive impairment, adjusting for known important variables such as age, years of education, CIMT, atrial fibrillation, cholesterol and hypertension, as well as potentially relevant variables including *APOE* ε4 status, cardiac indices such as stroke volume, renal function and HbA_1c_ levels.
- ***Hypothesis 3: comparing brain volume and cognitive performance in participants with increased left ventricular mass and specific left ventricular geometric patterns.*** Analysis will be similar to that for LVH, except that patients will be divided on the presence or absence of specific morphometric measures. That is, analyses will be performed at each time-point (cross-sectional), and also longitudinally, to investigate change across time. Repeated-measures t-tests will determine the extent of brain volume loss across the two time-points and compared between groups. Correlation with cognitive performance will be performed as above. Unadjusted differences for clinical variables between groups will be assessed by *t*-test. Difference in laboratory data and left ventricular systolic function will be adjusted for age and sex by use of ANCOVA. Differences in left ventricular structure and geometry will be adjusted for age, sex, BMI, and systolic blood pressure as their major established covariates with further adjustment for duration of hypertension. Fisher’s Exact Test and odds ratios will be used to test differences for categorical variables. Logistic regression analysis will be used to derive odds ratios adjusted for covariates. Multiple regression analyses will be performed to assess relation of cognitive and brain volume measures to higher left ventricular mass independent of established covariates and duration of hypertension.

## Discussion

The D2 study will aim to improve the understanding of two of the major causes of death, disability and reduced quality of life in our society: diabetes and dementia. In an ageing population, it is vitally important that we increase our knowledge of the relationships between cardiovascular disease and cognition. We have the means to identify a potentially treatable risk factor in the causation of cognitive impairment. The study will provide unique information on the relationship between LVH and dementia in type 2 diabetes, and whether cardiac structural and functional abnormalities contribute to increased brain atrophy and cognitive impairment. Brain volume changes may precede cognitive changes, providing a potential for early diagnosis and intervention. A large proportion of these patients may have associated or superadded Alzheimer’s pathology. These patients provide a high risk cohort requiring urgent intervention to prevent increasing dependence and disability. The identification of people at risk of cognitive impairment and dementia will allow for both secondary prevention and early intervention. With the advent of aggressive risk management strategies for cardiovascular disease, identification of these patients will allow them to be treated early in their disease course, ideally before they develop cognitive decline.

## Additional file

Additional file 1: D2 Study MRI: Data acquired on a Siemens 3 T Skyra scanner using a 64 Channel receive only head coil. (DOCX 12 kb)

## Abbreviations

3D: Three-dimension
APOE: Apolipoprotein E
ASE: American Society of Echocardiography
BMI: Body mass index
CIMT: Carotid intima media thickness
DWI: Diffusion weighted imaging
ECG: Electrocardiogram
eGFR: Estimated glomerular filtration rate
FLAIR: Fluid-attenuated inversion recovery
GAD-7: Generalized anxiety disorder questionnaire
LVH: Left ventricular hypertrophy
MET: Metabolic equivalents minute
MPRAGE: Magnetization-prepared rapid gradient-echo
MRI: Magnetic resonance imaging
PHQ-9: Patient health questionnaire
SWI: Susceptibility-weighted image
TTE: Transthoracic echocardiography

## References

[1] Biessels GJ, Staekenborg S, Brunner E, Brayne C, Scheltens P (2006). Risk of dementia in diabetes mellitus: a systematic review. Lancet Neurol.

[2] Van den Berg E, Kessels R, Kappelle L, De Haan E, Biessels G, Group UDES (2006). Type 2 diabetes, cognitive function and dementia: vascular and metabolic determinants. Drugs Today (Barc).

[3] Scheltens P, Fox N, Barkhof F, De Carli C (2002). Structural magnetic resonance imaging in the practical assessment of dementia: beyond exclusion. Lancet Neurol.

[4] Kivipelto M, Helkala EL, Laakso MP, Hanninen T, Hallikainen M, Alhainen K, Soininen H, Tuomilehto J, Nissinen A (2001). Midlife vascular risk factors and Alzheimer’s disease in later life: longitudinal, population based study. BMJ.

[5] Peila R, Rodriguez BL, Launer LJ (2002). Type 2 diabetes, APOE gene, and the risk for dementia and related pathologies The Honolulu-Asia Aging Study. Diabetes.

[6] Jack C, Shiung M, Weigand S, O’Brien P, Gunter J, Boeve B, Knopman D, Smith G, Ivnik R, Tangalos E (2005). Brain atrophy rates predict subsequent clinical conversion in normal elderly and amnestic MCI. Neurology.

[7] Jack CR, Lowe VJ, Weigand SD, Wiste HJ, Senjem ML, Knopman DS, Shiung MM, Gunter JL, Boeve BF, Kemp BJ (2009). Serial PIB and MRI in normal, mild cognitive impairment and Alzheimer’s disease: implications for sequence of pathological events in Alzheimer’s disease. Brain.

[8] Cardenas VA, Reed B, Chao LL, Chui H, Sanossian N, DeCarli CC, Mack W, Kramer J, Hodis HN, Yan M (2012). Associations among vascular risk factors, carotid atherosclerosis, and cortical volume and thickness in older adults. Stroke.

[9] van Elderen SG, de Roos A, de Craen AJ, Westendorp RG, Blauw GJ, Jukema JW, Bollen EL, Middelkoop HA, van Buchem MA, van der Grond J (2010). Progression of brain atrophy and cognitive decline in diabetes mellitus: a 3-year follow-up. Neurology.

[10] Launer LJ, Miller ME, Williamson JD, Lazar RM, Gerstein HC, Murray AM, Sullivan M, Horowitz KR, Ding J, Marcovina S (2011). Effects of intensive glucose lowering on brain structure and function in people with type 2 diabetes (ACCORD MIND): a randomised open-label substudy. Lancet Neurol.

[11] Christman AL, Matsushita K, Gottesman RF, Mosley T, Alonso A, Coresh J, Hill-Briggs F, Sharrett AR, Selvin E (2011). Glycated haemoglobin and cognitive decline: the Atherosclerosis Risk in Communities (ARIC) study. Diabetologia.

[12] Christman A, Matsushita K, Gottesman R, Mosley T, Alonso A, Coresh J, Hill-Briggs F, Sharrett A, Selvin E (2011). Glycated haemoglobin and cognitive decline: the Atherosclerosis Risk in Communities (ARIC) study. Diabetologia.

[13] Gerstein HC, Miller ME, Byington RP, Goff DC, Bigger JT, Buse JB, Cushman WC, Genuth S, Ismail-Beigi F, Action to Control Cardiovascular Risk in Diabetes Study G (2008). Effects of intensive glucose lowering in type 2 diabetes. N Engl J Med.

[14] Punthakee Z, Miller ME, Launer LJ, Williamson JD, Lazar RM, Cukierman-Yaffee T, Seaquist ER, Ismail-Beigi F, Sullivan MD, Lovato LC (2012). Poor cognitive function and risk of severe hypoglycemia in type 2 diabetes: post hoc epidemiologic analysis of the ACCORD trial. Diabetes Care.

[15] Murray AM, Hsu FC, Williamson JD, Bryan RN, Gerstein HC, Sullivan MD, Miller ME, Leng I, Lovato LL, Launer LJ (2017). ACCORDION MIND: results of the observational extension of the ACCORD MIND randomised trial. Diabetologia.

[16] Palmieri V, Bella JN, Arnett DK, Liu JE, Oberman A, Schuck MY, Kitzman DW, Hopkins PN, Morgan D, Rao DC (2001). Effect of type 2 diabetes mellitus on left ventricular geometry and systolic function in hypertensive subjects: Hypertension Genetic Epidemiology Network (HyperGEN) study. Circulation.

[17] Boonman-de Winter L, Rutten F, Cramer M, Landman M, Liem A, Rutten G, Hoes A (2012). High prevalence of previously unknown heart failure and left ventricular dysfunction in patients with type 2 diabetes. Diabetologia.

[18] Srivastava PM, Calafiore P, Macisaac RJ, Patel SK, Thomas MC, Jerums G, Burrell LM (2008). Prevalence and predictors of cardiac hypertrophy and dysfunction in patients with Type 2 diabetes. Clin Sci.

[19] Cuspidi C, Sala C, Negri F, Mancia G, Morganti A, Italian Society of H (2012). Prevalence of left-ventricular hypertrophy in hypertension: an updated review of echocardiographic studies. J Hum Hypertens.

[20] Bikkina M, Levy D, Evans JC, Larson MG, Benjamin EJ, Wolf PA, Castelli WP (1994). Left ventricular mass and risk of stroke in an elderly cohort. The Framingham Heart Study. JAMA.

[21] Bombelli M, Facchetti R, Carugo S, Madotto F, Arenare F, Quarti-Trevano F, Capra A, Giannattasio C, Dell’Oro R, Grassi G (2009). Left ventricular hypertrophy increases cardiovascular risk independently of in-office and out-of-office blood pressure values. J Hypertens.

[22] Filley CM, Brodtmann A (2011). Lacunes and cognitive decline: little things matter. Neurology.

[23] Eggermont LH, de Boer K, Muller M, Jaschke AC, Kamp O, Scherder EJ (2012). Cardiac disease and cognitive impairment: a systematic review. Heart.

[24] Vogels RL, Oosterman JM, van Harten B, Gouw AA, Schroeder-Tanka JM, Scheltens P, van der Flier WM, Weinstein HC (2007). Neuroimaging and correlates of cognitive function among patients with heart failure. Dement Geriatr Cogn Disord.

[25] Vogels RL, Scheltens P, Schroeder-Tanka JM, Weinstein HC (2007). Cognitive impairment in heart failure: a systematic review of the literature. Eur J Heart Fail.

[26] Vogels RL, van der Flier WM, van Harten B, Gouw AA, Scheltens P, Schroeder-Tanka JM, Weinstein HC (2007). Brain magnetic resonance imaging abnormalities in patients with heart failure. Eur J Heart Fail.

[27] Wendell CR, Zonderman AB, Metter EJ, Najjar SS, Waldstein SR (2009). Carotid intimal medial thickness predicts cognitive decline among adults without clinical vascular disease. Stroke.

[28] Scuteri A, Coluccia R, Castello L, Nevola E, Brancati AM, Volpe M (2009). Left ventricular mass increase is associated with cognitive decline and dementia in the elderly independently of blood pressure. Eur Heart J.

[29] Elias MF, Sullivan LM, Elias PK, D’Agostino RB, Wolf PA, Seshadri S, Au R, Benjamin EJ, Vasan RS (2007). Left ventricular mass, blood pressure, and lowered cognitive performance in the Framingham offspring. Hypertension.

[30] Jefferson AL, Himali JJ, Au R, Seshadri S, Decarli C, O’Donnell CJ, Wolf PA, Manning WJ, Beiser AS, Benjamin EJ (2011). Relation of left ventricular ejection fraction to cognitive aging (from the Framingham Heart Study). Am J Cardiol.

[31] Lang RM, Bierig M, Devereux RB, Flachskampf FA, Foster E, Pellikka PA, Picard MH, Roman MJ, Seward J, Shanewise JS (2005). Recommendations for chamber quantification: a report from the American Society of Echocardiography’s Guidelines and Standards Committee and the Chamber Quantification Writing Group, developed in conjunction with the European Association of Echocardiography, a branch of the European Society of Cardiology. JAm Soc Echocardiogr.

[32] Moneta GL, Edwards JM, Chitwood RW, Taylor LM, Lee RW, Cummings CA, Porter JM (1993). Correlation of North American Symptomatic Carotid Endarterectomy Trial (NASCET) angiographic definition of 70% to 99% internal carotid artery stenosis with duplex scanning. J Vasc Surg.

[33] Fischl B, Dale AM (2000). Measuring the thickness of the human cerebral cortex from magnetic resonance images. Proc Natl Acad Sci U S A.

[34] Pardoe H, Pell GS, Abbott DF, Berg AT, Jackson GD (2008). Multi-site voxel-based morphometry: methods and a feasibility demonstration with childhood absence epilepsy. Neuroimage.

[35] Pardoe HR, Pell GS, Abbott DF, Jackson GD (2009). Hippocampal volume assessment in temporal lobe epilepsy: How good is automated segmentation?. Epilepsia.

[36] Brodtmann A, Werden E, Pardoe H, Li Q, Jackson G, Donnan G, Cowie T, Bradshaw J, Darby D, Cumming T (2014). Charting cognitive and volumetric trajectories after stroke: protocol for the Cognition And Neocortical Volume After Stroke (CANVAS) study. Int J Stroke.

[37] American Psychiatric Association. Diagnostic and statistical manual of mental disorders. 5th ed. Arlington: Am Psychiatr Assoc; 2013.

[38] Kim J, Tanabe K, Yokoyama N, Zempo H, Kuno S (2013). Objectively measured light-intensity lifestyle activity and sedentary time are independently associated with metabolic syndrome: a cross-sectional study of Japanese adults. Int J Behav Nutr Phys Act.

[39] St-Onge M, Mignault D, Allison DB, Rabasa-Lhoret R (2007). Evaluation of a portable device to measure daily energy expenditure in free-living adults. Am J Clin Nutr.

[40] Chen K, Langbaum JB, Fleisher AS, Ayutyanont N, Reschke C, Lee W, Liu X, Bandy D, Alexander GE, Thompson PM (2010). Twelve-month metabolic declines in probable Alzheimer’s disease and amnestic mild cognitive impairment assessed using an empirically pre-defined statistical region-of-interest: findings from the Alzheimer’s Disease Neuroimaging Initiative. Neuroimage.

[41] Hua X, Lee S, Hibar DP, Yanovsky I, Leow AD, Toga AW, Jack CR, Bernstein MA, Reiman EM, Harvey DJ (2010). Mapping Alzheimer’s disease progression in 1309 MRI scans: power estimates for different inter-scan intervals. Neuroimage.

[42] Leow AD, Yanovsky I, Parikshak N, Hua X, Lee S, Toga AW, Jack CR, Bernstein MA, Britson PJ, Gunter JL (2009). Alzheimer’s disease neuroimaging initiative: a one-year follow up study using tensor-based morphometry correlating degenerative rates, biomarkers and cognition. Neuroimage.

[43] Brodtmann A, Puce A, Darby D, Donnan G (2009). Regional fMRI brain activation does correlate with global brain volume. Brain Res.

[44] Nitkunan A, Lanfranconi S, Charlton RA, Barrick TR, Markus HS (2011). Brain atrophy and cerebral small vessel disease: a prospective follow-up study. Stroke.

[45] Nasreddine ZS, Phillips NA, Bédirian V, Charbonneau S, Whitehead V, Collin I, Cummings JL, Chertkow H (2005). The Montreal Cognitive Assessment, MoCA: a brief screening tool for mild cognitive impairment. J Am Geriatr Soc.

[46] Morris JC (1993). The Clinical Dementia Rating (CDR): current version and scoring rules. Neurology.

[47] Nelson HE, Willison J (1991). National Adult Reading Test (NART): Nfer-Nelson Windsor.

[48] Tombaugh TN (2004). Trail Making Test A and B: normative data stratified by age and education. Arch Clin Neuropsychol.

[49] Wechsler D (2014). Wechsler Adult Intelligence Scale–Fourth Edition (WAIS–IV).

[50] Maruff P, Lim YY, Darby D, Ellis KA, Pietrzak RH, Snyder PJ, Bush AI, Szoeke C, Schembri A, Ames D (2013). Clinical utility of the cogstate brief battery in identifying cognitive impairment in mild cognitive impairment and Alzheimer’s disease. BMC Pharmacol Toxicol.

[51] Meyers JE, Meyers KR (1995). Rey complex figure test under four different administration procedures. Clin Neuropsychol.

[52] Royall DR, Cordes JA, Polk M (1998). CLOX: an executive clock drawing task. J Neurol Neurosurg Psychiatry.

[53] Tombaugh TN, Kozak J, Rees L (1999). Normative data stratified by age and education for two measures of verbal fluency: FAS and animal naming. Arch Clin Neuropsychol.

[54] Saxton J, Ratcliff G, Munro CA, Coffey EC, Becker JT, Fried L, Kuller L (2000). Normative data on the Boston Naming Test and two equivalent 30-item short forms. Clin Neuropsychol.

[55] Schmidt M. Rey Auditory and Verbal Learning Test: A handbook: Western Psychological Services Los Angeles; 1996.

