# Supplementary material for: Does left ventricular hypertrophy affect cognition and brain structural integrity in type 2 diabetes? Study design and rationale of the Diabetes and Dementia (D2) study
Source: BMC Endocr Disord. 2017 Apr 7;17:24. doi: 10.1186/s12902-017-0173-7 (PMC5384138; doi:10.1186/s12902-017-0173-7)
Supplement: Additional file 1: — D2 Study MRI: Data acquired on a Siemens 3 T Skyra scanner using a 64 Channel receive only head coil. (DOCX 12 kb) [file 12902_2017_173_MOESM1_ESM.docx]

**Additional file 1**

D2 Study MRI: Data acquired on a Siemens 3T Skyra scanner using a 64 Channel receive only head coil.

**T1 3D MPRAGE**

FOV = 256mm

Slices per Slab = 176

Voxel Size = 1mm isotropic

TR = 1900ms

TE = 2.43ms

Flip Angle = 9 degrees

Bandwidth = 180Hz/Px

**T2 FLAIR 3D**

FOV = 256mm

Slices per Slab = 176

Voxel Size = 1mm isotropic

TR = 6000ms

TE = 390ms

TI = 2100ms

Bandwidth = 781Hz/Px

Turbofactor = 278

Echo Train Duration = 862ms

**T2 3D**

FOV = 256mm

Slices per Slab = 182

Voxel Size = 1mm isotropic

TR = 3200ms

TE = 412ms

Bandwidth = 723Hz/Px

Turbofactor = 282

Echo Train Duration = 912ms

**SWI**

FOV = 224mm

Voxel Size = 0.5x0.5x2mm

Slices per Slab = 72

TR = 26ms

TE = 19.70ms

Flip Angle = 15 degrees

Bandwidth = 200Hz/Px

**DWI**

FOV = 240mm

*b* value = 3000ms^2^

DWI directions = 64 + 7xb0 images

Voxel Size = 2.5mm isotropic

Slices = 60

TR = 8400ms

TE = 110ms

Bandwidth = 2368 Hz/Px

**GRE field mapping**

FOV = 216mm

Voxel Size = 3mm isotropic

Slices = 44

TR = 500ms

TE1 = 5.19ms, TE2 = 7.65ms

Flip Angle = 60 degrees

Bandwidth = 260 Hz/Px

**BOLD 7 minute free run (EPI*)**

FOV = 216mm

Voxel Size = 3mm isotropic

Slices = 44

TR = 3000ms

TE = 30ms

Flip Angle = 90 degrees

Bandwidth = 1102 Hz/Px
